# Ethics of neuroimaging after serious brain injury

**DOI:** 10.1186/1472-6939-15-41

**Published:** 2014-05-20

**Authors:** Charles Weijer, Andrew Peterson, Fiona Webster, Mackenzie Graham, Damian Cruse, Davinia Fernández-Espejo, Teneille Gofton, Laura E Gonzalez-Lara, Andrea Lazosky, Lorina Naci, Loretta Norton, Kathy Speechley, Bryan Young, Adrian M Owen

**Affiliations:** 1 Rotman Institute of Philosophy, Western University, London, ON, N6A 5B8, Canada; 2 Brain and Mind Institute, Western University, London, ON, N6A 5B7, Canada; 3 Department of Medicine, London Health Sciences Centre–University Hospital, London, ON, N6A 5A5, Canada; 4 Department of Epidemiology and Biostatistics, Western University, London, ON, N6A 5C1, Canada; 5 Department of Family and Community Medicine, University of Toronto, Toronto, ON, M5G 1V7, Canada; 6 Department of Clinical Neurological Sciences, London Health Sciences Centre–University Hospital, London, ON, N6A 5A5, Canada; 7 Department of Psychiatry, London Health Sciences Centre–Victoria Hospital, London, ON, N6A 5W9, Canada; 8 Department of Pediatrics, Children’s Hospital of Western Ontario, London, ON, N6C 2V5, Canada

**Keywords:** Ethics, Brain injury, Vegetative state, Unresponsive wakefulness syndrome, Minimally conscious state, Functional magnetic resonance imaging, Electroencephalography, Decision making capacity, Informed consent, Quality of life, End of life care

## Abstract

**Background:**

Patient outcome after serious brain injury is highly variable. Following a period of coma, some patients recover while others progress into a vegetative state (unresponsive wakefulness syndrome) or minimally conscious state. In both cases, assessment is difficult and misdiagnosis may be as high as 43%. Recent advances in neuroimaging suggest a solution. Both functional magnetic resonance imaging and electroencephalography have been used to detect residual cognitive function in vegetative and minimally conscious patients. Neuroimaging may improve diagnosis and prognostication. These techniques are beginning to be applied to comatose patients soon after injury. Evidence of preserved cognitive function may predict recovery, and this information would help families and health providers. Complex ethical issues arise due to the vulnerability of patients and families, difficulties interpreting negative results, restriction of communication to “yes” or “no” answers, and cost. We seek to investigate ethical issues in the use of neuroimaging in behaviorally nonresponsive patients who have suffered serious brain injury. The objectives of this research are to: (1) create an approach to capacity assessment using neuroimaging; (2) develop an ethics of welfare framework to guide considerations of quality of life; (3) explore the impact of neuroimaging on families; and, (4) analyze the ethics of the use of neuroimaging in comatose patients.

**Methods/Design:**

Our research program encompasses four projects and uses a mixed methods approach. Project 1 asks whether decision making capacity can be assessed in behaviorally nonresponsive patients. We will specify cognitive functions required for capacity and detail their assessment. Further, we will develop and pilot a series of scenarios and questions suitable for assessing capacity. Project 2 examines the ethics of welfare as a guide for neuroimaging. It grounds an obligation to explore patients’ interests, and we explore conceptual issues in the development of a quality of life instrument adapted for neuroimaging. Project 3 will use grounded theory interviews to document families’ understanding of the patient’s condition, expectations of neuroimaging, and the impact of the results of neuroimaging. Project 4 will provide an ethical analysis of neuroimaging to investigate residual cognitive function in comatose patients within days of serious brain injury.

## Background

Improvements in intensive care have led to an increased survival rate following serious brain injury, but patient outcome is highly variable. Following a period of coma, some patients go on to make a good recovery, while others progress into a vegetative state (unresponsive wakefulness syndrome) or minimally conscious state [1]. Patients in a vegetative state are awake but have no awareness of self or environment, while patients in a minimally conscious state show inconsistent evidence of awareness. In both cases, assessment is very difficult and depends on subjective interpretation of observed behavior. Indeed, the misdiagnosis rate may be as high as 43% in these groups [2-4].

Recent advances in neuroimaging suggest a solution. Both functional magnetic resonance imaging (fMRI) and electroencephalography (EEG) have been used to detect residual cognitive function and even conscious awareness in vegetative and minimally conscious patients [5-13]. Neuroimaging may improve diagnosis and prognostication in these patients. Indeed, neuroimaging has shown that a subset of this patient group (17–19%), who consistently satisfy the behavioral diagnostic criteria of the vegetative state, retain conscious awareness undetectable through bedside clinical examination [6,7,14]. Additionally, in at least 3 reported cases, neuroimaging has been used to communicate with patients diagnosed as vegetative or minimally conscious, raising the prospect of involving them in decisions regarding their care [6,12,13]. These techniques are now being studied in comatose patients soon after serious brain injury. Evidence of preserved cognitive function in these patients may predict recovery, and, if so, this information will be valuable to families weighing the continuation—or withdrawal—of life-sustaining therapy.

Despite the promise of neuroimaging after serious brain injury, complex ethical issues must be addressed before the technology is broadly adopted. These ethical issues arise due to difficulties interpreting negative neuroimaging results, restriction of communication to “yes” or “no” answers, the vulnerability of patients and families, and the cost of fMRI. This research program seeks to investigate ethical issues in the use of neuroimaging in behaviorally nonresponsive patients who have suffered serious brain injury. Both the research team and research program are closely integrated with a Canadian Institutes of Health Research (CIHR) funded project using fMRI and EEG to measure residual cognitive function in this patient population.

Our research program encompasses four main projects: (1) the assessment of decision making capacity in behaviorally nonresponsive patients; (2) the ethics of welfare as a moral framework for behaviorally nonresponsive patients who retain covert awareness; (3) the impact of neuroimaging on families of patients with a serious brain injury; and (4) an analysis of ethical issues in the use of neuroimaging in comatose patients within days of serious brain injury. Our research program uses a mixed methods approach, employing ethical analysis and a variety of empirical techniques, including fMRI studies of healthy volunteers, quality of life instrument development, and interviews of families.

### Neuroimaging in vegetative and minimally conscious patients

To date, functional neuroimaging studies of patients following serious brain injury have focused on vegetative or minimally conscious patients more than one year after injury. In the vegetative state, patients exhibit regular sleep-wake cycles, but show no awareness of self or their environment [15,16]. The vegetative state can result from traumatic and non-traumatic brain injuries, metabolic or degenerative disorders, and congenital malformations of the central nervous system [16]. Recovery of cognitive function is unlikely more than 12 months after trauma to the brain, and rare more than 3 months following non-traumatic events [16]. Once medically stable, vegetative patients are typically cared for in a chronic care hospital or in the family home with outside support.

Diagnosis of the vegetative state relies upon a variety of bedside clinical examinations [17-19]. A diagnosis is made after repeated examinations have yielded no evidence of sustained, reproducible, purposeful, or voluntary behavioral response to visual, auditory, tactile, or painful stimuli [16]. The minimally conscious state is a relatively new diagnostic category, and describes patients who show inconsistent but reproducible evidence of awareness [20]. As we have noted, assessment of vegetative and minimally conscious patients is difficult and the error rate in diagnosis may be as high as 43% [2-4].

Recent developments in neuroimaging have the potential to improve the accuracy of diagnosis in vegetative and minimally conscious patients [5-12]. The majority of neuroimaging methods to detect covert awareness use willful modulation of brain activity as a proxy for behavioral command following. In the first reported use of the technique, a 23-year old patient, who had been diagnosed as vegetative for one year, imagined playing tennis and moving from room to room in her house when instructed to do so [5]. The hemodynamic changes in her brain were unique to volitionally imagining these activities, and her response was indistinguishable from healthy controls [21].

This mental imagery paradigm was then used in 54 patients diagnosed as vegetative or minimally conscious [6]. Four patients (17%) clinically diagnosed as vegetative could modulate their brain activity in response to commands, thereby demonstrating covert awareness. Moreover, investigators extended the mental imagery technique to permit communication [6]. Patients were asked a question and instructed to imagine playing tennis if their answer was “yes”, and to imagine walking from room to room in their house if their answer was “no”. One patient, whose vegetative diagnosis was confirmed over a five-year period, correctly answered several autobiographical questions pertaining to his father, siblings, and the last country visited prior to injury [6]. Most recently, a patient in London, Ontario, who had been diagnosed as vegetative for 12 years, was asked a series of questions, including “are you in physical pain?” [13]. The patient was able to answer the questions posed and this exchange illustrates the potential clinical utility of neuroimaging communication in this patient population.

Recent technical developments have sought to improve the efficiency of fMRI and utilize other neuroimaging modalities. One approach uses a similar fMRI mental imagery paradigm, yet requires the patient to merely activate the brain’s attention network, decreasing the amount of imaging time required to assess command following and communication [12,22]. Additionally, recent innovations in EEG have aimed to develop a less expensive and portable imaging technique that mitigates the restricted access and cost of fMRI scanning time [7,8,10]. Because patients do not need to be transported to a hospital or fMRI research facility for EEG examination, the physical stress on the patient is decreased and accessibility is broadened.

In a study funded by CIHR (2013–2018) and running in parallel with our proposed research program, members of our research team will use neuroimaging to build a detailed map of residual cognitive function in 125 vegetative or minimally conscious patients following serious brain injury—the largest cohort ever studied. So far data have been collected on 24 patients: patients are young (35 years old on average at the time of neuroimaging assessment) and predominantly male (71%); a slight majority are diagnosed as vegetative (58%); the mechanism of injury is evenly divided between traumatic and non-traumatic causes and the median time since injury is 5 years (range 1–19 years at the time of neuroimaging assessment); 16 patients are cared for in a hospital or a long term care facility, while 8 are cared for at home. The study seeks to improve diagnostic accuracy in these patients and identify new and objective prognostic markers. Patients will undergo a broad battery of neuroimaging tests using fMRI and EEG to document preserved sound perception, speech perception and comprehension, visual perception, attention, memory, command following, and communication abilities. This information will help refine the patient’s diagnosis by documenting preserved function. Preserved cognitive function may also predict better patient outcome. In one small study in this patient population, speech perception and comprehension correlated with signs of recovery six months after neuroimaging [23].

### Neuroimaging and comatose patients

Recently, these neuroimaging techniques have been extended to explore residual cognitive function in comatose patients within days of serious brain injury. After a serious brain injury, patients may be comatose for 2 to 4 weeks. Comatose patients are unarousably unconscious, do not open their eyes, and only exhibit reflex responses to stimulation [1]. Some patients go on to make a good recovery, while others progress into vegetative or minimally conscious states. Currently, prognosis in coma is assessed using clinical examination, structural neuroimaging, and somatosensory evoked potentials (SSEPs: testing for intact pathways between the median nerve and the sensory cortex). After cardiac arrest, outcome is very poor in patients with absent pupillary reflexes, absent or extensor motor response to pain, or bilaterally absent SSEPs [24]. After traumatic brain injury, additional predictors of poor outcome include structural abnormalities (e.g., intraventricular blood or subarachnoid hemorrhage) [25]. Despite these indicators, prognostication involves subjective judgment and experience on the part of the neurologist.

The majority of deaths subsequent to serious brain injury follow a decision to withdraw life-sustaining therapy (LST). A recent study of Canadian trauma centers revealed a mortality rate of 32% following serious brain injury and found that 70% of these deaths were associated with the withdrawal of LST [26]. Many decisions to withdraw LST occurred very early, with one half of deaths within 72 hours of injury. According to the authors, “[i]n some instances, this may be too early for accurate neuroprognostication” [26]. The study further documented considerable variation in mortality between hospitals that persisted after adjustment for patient risk factors. The authors concluded that “[t]his raises the concern that differences in mortality between centres may be partly due to variation in physicians’ perceptions of long-term prognosis” [26]. Families of patients with serious brain injury may perceive a “window of opportunity” for the withdrawal of care to avoid undesirable patient outcomes, extending from the time of injury to the point when the patient is no longer dependent on mechanical ventilation [27]. Families considering withdrawal of LSTs need high levels of communication, including access to accurate prognostic information [28].

In the CIHR-funded study (2013–2018) referred to above, members of our research team will use neuroimaging to build a detailed map of residual cognitive function in an additional 125 patients who are comatose following serious brain injury. They will use techniques developed for patients in vegetative and minimally conscious states and apply them to comatose patients in the intensive care unit within days of injury. So far data has been collected on 12 patients: the mean age is 50 years old and patients are predominantly male (75%); all were comatose and mechanically ventilated in an intensive care unit; the median time since injury is 19.5 days. The data indicate that fMRI and EEG can be applied in this setting and, in a proportion of comatose patients, covert cognitive function can be detected. Improving access to accurate and timely prognostic information after serious brain injury holds the prospect of improving treatment decisions and patient care.

### Ethical and scientific controversies

Despite the promise of neuroimaging after serious brain injury, the scientific and ethics communities reacted to early findings with skepticism. After the first report of covert awareness in a vegetative patient [5], critics questioned whether fMRI findings were, in fact, evidence of command following [29,30]. It was argued that verbal stimuli can produce spontaneous neural activation, and thus the observed hemodynamic changes could merely be unconscious speech processing. More recently, others have questioned EEG studies in behaviorally nonresponsive patients [7]. Critics argued that the statistical methods employed were not sufficiently conservative, thereby risking false positive results [31].

We contend that fMRI responses to the mental imagery task do not represent a reflex or unconscious speech processing [14,32]. Hemodynamic activity is observed in areas of the brain associated with motor activity and spatial navigation that are distinct from the auditory cortex. Further, neural activation is sustained for 30 second periods until the patient is instructed to “relax”, and as a result is unlikely to be linked to unconscious speech processing. Moreover, the mental imagery paradigm has been used to communicate with at least 3 patients [6,12,13]. Patients have correctly answered biographical questions, which cannot be explained by appeal to unconscious speech processing.

Concerns raised about the validity of statistical methods used in our EEG research have been addressed in part by appealing to fMRI data as corroborating evidence [33]. Moreover, our EEG technique detailed in the *Lancet* classifies 75% of healthy participants as aware. In contrast, statistical methods proposed by critics produce variable results on identical control data [31]. Because healthy participants are known to be conscious, it stands to reason that statistical methods that are unable to detect awareness in this group may not detect covert awareness, when it exists, in behaviourally nonresponsive patients [33].

In the ethics literature, a variety of concerns have been raised about neuroimaging after serious brain injury. Much of the discussion has focused on the possibility of behaviorally nonresponsive patients participating in decisions regarding their own care. Indeed, if a patient is able to communicate via neuroimaging, one can imagine a number of questions that might be posed regarding care. For example, one might ask whether a patient wishes to be resuscitated in the event of a cardiac arrest. Critics point out—correctly we think—that before such questions are posed, patients’ decision making capacity must first be established. Given that neuroimaging communication only provides for “yes” or “no” responses to questions and patients cannot ask questions of their own, some have argued that it is not possible to evaluate decision making capacity [34-37]. If we cannot assess decision making capacity, it is suggested that the clinical utility of neuroimaging is undercut [34-37].

Others ask “if we have evidence of consciousness in a patient previously thought to be in a vegetative state, is it permissible to withdraw life-sustaining treatment?” [38]. The moral permissibility of withdrawal of LSTs, including artificial nutrition and hydration, from patients in vegetative state is widely accepted. Evidence of consciousness, however, is thought by some to be a reason not to withdraw LSTs. Indeed, in several well-publicized legal cases it has been suggested that vegetative patients undergo neuroimaging.

Some have been critical of this view and have said that the discovery of covert consciousness in a vegetative patient does not provide us with a clear reason to keep the patient alive. They argue that “[i]f such patients suffer they can be harmed by continuing treatment; there may be stronger reasons in terms of non-maleficence and the best interests of the patient to allow them to die” [38]. If correct, this would mitigate the utility of neuroimaging in ethical and legal controversies regarding the withdrawal of care.

Finally, it has been argued that the clinical application of neuroimaging may have a negative impact on the emotional well-being or finances of the families of patients with serious brain injuries [34,35,39]. This derives in part from the lack of knowledge regarding the precise correlation between physical findings at the bedside exam, and neuroimaging results. Because we know so little about consciousness and its precise relation to neural mechanisms, it is argued that we must proceed with caution before clinical use of neuroimaging in patients after serious brain injury is endorsed [35,40].

### Study objectives

The overarching goal of this project is to investigate ethical issues in the use of neuroimaging in behaviorally nonresponsive patients who have suffered serious brain injury. We will build a lasting collaboration between philosophers, physicians, and neuroscientists that draws upon the strengths of each discipline, produces significant contributions to the scholarly literature, advances ethical practice in neuroimaging, and ultimately improves the care of patients with serious brain injuries. Specific objectives are to:

1. Create a conceptual framework for capacity assessment in behaviorally nonresponsive patients using neuroimaging and demonstrate its feasibility in healthy controls;

2. Develop an ethics of welfare framework to guide the use of neuroimaging in behaviorally nonresponsive patients and apply it to considerations of quality of life;

3. Explore the impact of neuroimaging on the families of behaviorally nonresponsive patients; and,

4. Provide the first sustained ethical analysis of the use of neuroimaging to detect residual cognitive function in comatose patients.

## Methods/Design

Our research program uses a mixed methods approach, employing ethical analysis and a variety of empirical techniques, including fMRI studies of healthy volunteers, quality of life instrument development, and interviews of families. Ethical analysis in bioethics is not amenable to the degree of a priori methodological specification that is expected of empirical research. Statistical rigor and reproducibility are indispensable features of science, necessitating the clear and up-front statement of hypotheses and experimental methods. High-quality ethical analysis, on the other hand, relies neither on statistical rigor nor on reproducibility. Rather, it begins with the articulation of clear and important questions and is realized in the construction of sound arguments in peer-reviewed publications and policy reports. For each question, an extensive review of the scholarly literature will document and critically analyze arguments proffered for and against particular positions. Where gaps exist, we will develop our own ethical arguments. The ethical analysis will then seek to synthesize foundational documents, regulations, and existing and novel arguments into a coherent position. Where disagreement among the various sources cannot be resolved by ethical analysis, the details of the dispute will be documented. Sound ethical analysis relies upon a foundation of reliable data. Accordingly, we plan to conduct several empirical studies to inform, complement, and enrich planned ethical analyses. Detailed methods for the fMRI studies of healthy volunteers, quality of life instrument development, and family interviews are provided in the project descriptions below.

### Project 1: Assessing decision making capacity in behaviorally nonresponsive patients

Providing informed consent for medical treatment requires the capacity to make autonomous decisions [41-44]. Given that a proportion of behaviorally nonresponsive patients with severe brain injury can now communicate using neuroimaging, it is timely to ask whether they can participate in decisions regarding their own medical care. As we explain above, a number of authors have argued that decision making capacity cannot be assessed using neuroimaging [35-37]. We have recently argued that capacity assessment in such patients is—in principle at least—possible [45].

Our argument proceeds by analyzing the MacArthur Competency Assessment Tool [43] and decomposing the standard elements of capacity—understanding, appreciation, reasoning, and communication—into constitutive cognitive functions measurable through neuroimaging [45]. By reducing the elements of capacity to components that can be evaluated using neuroimaging, we argue that a decisionally capable yet behaviorally nonresponsive patient may demonstrate decision making capacity through neuroimaging. In practice, this procedure may utilize a battery of validated neuropsychological instruments adapted for neuroimaging communication, which probe these constitutive components. We are careful to point out that the threshold for decision making capacity must be calibrated to the stakes of the treatment decision. It may be that capacity assessment using neuroimaging turns out to be applicable only to low and medium stakes treatment decisions, and is inappropriate for high stakes (e.g., end of life) decisions.

Having set out this provisional conceptual framework for capacity assessment in behaviorally nonresponsive patients using neuroimaging, further work is required. We envision that the assessment of decision making capacity in these patients will involve a two-step process. First, it must be shown that a patient possesses the cognitive functions necessary to make *any decision* regarding care. Second, once this has been established, it must be shown that the patient is capable of making the *particular decision* at stake. In order to address the first step, we aim to (1) provide a list of cognitive functions that may defensibly constitute understanding, appreciation, and reasoning, and (2) detail the assessment of these cognitive functions using neuroimaging. In order to address the second step, we will (3) develop a series of questions suitable for neuroimaging communication to assess a patient’s capacity to make a particular decision, and (4) demonstrate the feasibility of the neuroimaging assessment of decision making capacity in healthy controls. Here we set out two subprojects to achieve these goals.

#### ***Subproject 1a: Cognitive functions underpinning decision making capacity***

In our first subproject, we will specify constitutive cognitive functions necessary for decision making capacity. Decision making capacity is comprised of understanding, appreciation, reasoning, and communication. Since it has been demonstrated that neuroimaging can be used to communicate a choice, we will focus our analysis on the remaining elements. We will argue that each of these elements is underpinned by a complex set of cognitive functions that constitute the higher order mental operations referred to as understanding, appreciation, and reasoning. For example, standard neuropsychiatric instruments measure the appreciation of medical information as a product of consequential reasoning [43]. A patient appreciates the choice at hand if he or she can identify the consequences both of choosing and refusing treatment. Underlying consequential reasoning is a set of simpler cognitive functions that, taken together, allow a patient to appreciate information. These functions may include the ability to localize one’s self in space and time, the ability to relate medical information to oneself as opposed to another, and the ability to recognize temporal ordering in the environment. By utilizing an extensive literature on psychometrics, particularly those that probe abstract reasoning abilities (e.g., Wechsler Intelligence Scale, Woodcock Johnson Psychoeducational Scale), we will develop a model of the cognitive functions necessary for appreciation, understanding, and reasoning in the clinical context. Associating the cognitive profiles with neuroimaging techniques to assess the cognitive functions will provide a detailed approach to ascertain whether a patient possesses the cognitive functions needed to make *any decision* regarding care.

#### ***Subproject 1b: Demonstrating the feasibility of decision making capacity assessment using neuroimaging***

Decision making capacity is decision specific [41]. Once it has been established that a patient possesses the requisite cognitive functions outlined above, it remains to be shown that he or she is capable of making a particular decision in the clinical context. In this subproject, we seek to develop a series of questions suitable for neuroimaging communication to assess a patient’s capacity to make particular decisions, and to demonstrate the feasibility of this approach by studying healthy volunteers. We will develop a series of medical scenarios appropriate for healthy volunteers that correspond to low, medium, and high stakes medical decisions. Using criteria for capacity in the Ontario Health Care Consent Act (1996) [46], we will construct a series of questions for each scenario that evaluates the participant’s understanding and appreciation of the decision. We aim to answer two related questions with this study: (1) How many questions evaluating decision making capacity can be posed in one neuroimaging session? (2) Can healthy volunteers reliably demonstrate that they possess the capacity to make decisions regarding care through neuroimaging?

To investigate these questions, we will utilize recent work by Naci and colleagues [12,22], which details a method of neuroimaging communication using a participant’s attention network. This method reduces the scanning time needed to ask questions relative to previous techniques. In 92% of healthy participants, this method accurately decoded answers within 5.6 minutes [22]. This suggests that it is possible for a healthy participant to answer 10 questions in a 60-minute scanning session with a high degree of accuracy.

Ensuring the reliability of effects at the single-subject level is an important criterion for successful application of this method to testing individual patients in future studies. Following previous work on single subject fMRI techniques in healthy participants [21,22], our study will recruit 20 healthy volunteers. Volunteers will be native English speakers between the ages of 18 and 60, have no history of neurologic or psychiatric illness and normal hearing. Data will be acquired using a 3-T Siemens Tim Trio system, with a 32-channel head coil, at the Robarts Research Institute in London, Ontario. Data analysis will be performed using the Statistical Parametric Mapping 8 (Wellcome Institute of Cognitive Neurology, http://www.fil.ion.ucl.ac.uk/spm/software/spm8/), Automatic Analysis software (http://www.cusacklab.org), and the MarsBar SPM toolbox (http://marsbar.sourceforge.net/).

Because this neuroimaging method [12,22] utilizes functional activation of the attention network as a proxy for behavioural command following, a high level of confidence in the results is necessary to avoid type I errors. To ensure conservative testing, the results of each communication scan will be tested against a priori hypotheses derived from an initial localizer scan acquired for each individual participant. This localizer scan will map an individual’s attention network, and determine whether all or parts of the canonical attention network, observed at the group-level, are expected to be activated during selective attention in a functional communication scan for that individual participant. Whole-brain analysis of the fMRI data from the localizer scan will be used to determine significant brain activity, and, thus, localize the attention network in each participant. Based on the brain activity peaks within each participant’s attention network, two regions of interest (ROIs) will be determined with the MarsBar toolbox, to analyze brain-responses during the communication scans. Confining analysis within these independently defined and subject-specific ROIs will enhance sensitivity to true positive effects at the single-subject level.

To test a participant’s decision-making capacity in the communication scans brief yet realistic medical scenarios will be read to participants as they lie in the fMRI scanner. We will ask a set of 10 questions to assess the participant’s understanding and appreciation of the treatment choice in each scenario. Binary (“yes” or “no”) answers based on the fMRI data will be compared to the volunteer’s verbally reported answers. Scores of greater than or equal to 9 correct answers will be considered a “success”. Decoding accuracy at the group level will be determined with a binomial test. With a sample size of 20 participants, statistical significance (p < 0.05) will be achieved if 14 or more participants succeed. If the true probability of success is 80% [22], 20 participants provide a power of 80%. If successful, this study will be the first demonstration of capacity assessment using neuroimaging-based communication methods.

### Project 2: The ethics of welfare as a moral framework for behaviorally nonresponsive patients who retain awareness

The assessment of decision making capacity is an important problem after serious brain injury [45]. However, clinical decision making questions (e.g., “Do you give us permission to administer a narcotic to treat your pain?”) are only one type of question that might be asked of these patients. Another kind of question seeks reports of subjective experience (e.g., “Are you in pain?”), which have central relevance to the daily lives of patients. While questions related to medical decision making are grounded in respect for autonomy, questions exploring the subjective experience of patients may be viewed through the lens of the ethics of welfare. In this project, we examine the ethics of welfare as a moral framework to guide the use of neuroimaging in behaviorally nonresponsive patients after serious brain injury.

According to the ethics of welfare, for any moral decision, we must give due consideration to the interests of those affected by our actions [47]. The fact that an individual is sentient—capable of experiencing pain or pleasure—gives him or her a specific set of interests that must be taken into account in ethical decision making. Accordingly, it would be morally wrong to ignore these interests or to treat them as less important than our own [47-49]. It is important to note that the moral weight of interests does not depend on the possession of rational capacities; it is just as wrong to let a young child suffer needlessly as it is to allow an autonomous adult to suffer needlessly. As the ethics of welfare does not depend on the presence of clearly functioning rational faculties, it may be a useful approach in behaviorally nonresponsive patients after serious brain injury if decision making capacity cannot be verified.

The ethics of welfare helps direct the questions that might be asked of patients with serious brain injury. Communication through neuroimaging may allow some patients to express their interests, allowing families and health providers to take appropriate action. Indeed, just knowing that a patient is capable of experiencing pain and pleasure is sufficient grounds to take these interests seriously and may influence care (e.g., administering analgesia prior to a potentially painful intervention). To develop the ethics of welfare framework we will: (1) argue that patients after serious brain injury who demonstrate signs of conscious awareness should be regarded as sentient; (2) explore the implications of an ethics of welfare approach to end of life decisions in behaviorally nonresponsive patients; and (3) develop quality of life measures that can be administered through neuroimaging communication.

#### ***Subproject 2a: Conscious awareness and sentience in behaviorally nonresponsive patients***

Patients who successfully complete the mental imagery paradigm have demonstrated that they have a number of preserved cognitive functions, including auditory processing, speech processing, short-term memory, and rudimentary executive function. Does this imply that they are also sentient? In this subproject, we will pursue and develop three lines of argument to support the relationship between conscious awareness and sentience. The first line of argumentation appeals to the neuroanatomical basis of awareness and sentience. If the functional integrity of neural structures required for sentience is a subset of those required for awareness, then identification of awareness is evidence of sentience as well. A second line of argument considers the role of indirect markers of pain, including biomarkers (e.g., elevated heart rate or blood pressure) or neuroimaging data equivalent to healthy controls in “pain states” [50-52]. Third, in the subset of patients with whom communication is possible, the ability to experience pain or pleasure can be tested to allow patients to volitionally report that a stimulus is painful.

#### ***Subproject 2b: The ethics of welfare and end-of-life decisions in behaviorally nonresponsive patients***

Some have argued that the detection of sentience in behaviorally nonresponsive patients provides a strong reason to withdraw LST [36,38,53]. It is suggested that sentience combined with profound neurological disability may lead to terrible suffering. In such cases, there is an imperative to reduce suffering by allowing patients to die. While this view is consistent with the ethics of welfare, we will dispute the authors’ assumption that these patients are living lives of terrible suffering. While it is true that some members of the public and physicians treating patients in vegetative and minimally conscious states have strong intuitions about their subjective experiences [54,55], we will argue that intuition may provide poor insight into what life is like after a serious brain injury. Indeed, evidence of the subjective experiences of patients with a comparable disorder, locked-in syndrome, seems to contradict these intuitions. In locked-in syndrome, otherwise cognitively intact patients are unable to move their limbs or speak, but they may be able to communicate through vertical eye movements. In one study of the quality of life of patients with locked-in syndrome, a majority professed happiness, while only a minority were miserable [56]. In another study, the self-reported scores of locked-in patients for mental health, general health, and bodily pain were close to those of healthy controls [57]. We will argue that an ethics of welfare requires that—where possible—we communicate with patients after serious brain injury and allow them to report their own subjective quality of life.

#### ***Subproject 2c: Measuring quality of life in behaviorally nonresponsive patients***

In this project we will undertake the preliminary development of quality of life (QoL) instruments for use in behaviorally nonresponsive patients after serious brain injury. Overall QoL is “a broad construct, contributed to by many aspects of life of which health is only one”; health-related QoL, on the other hand, is a narrower construct, and focuses on the assessment of the individual’s subjective health experience [58]. The application of QoL instruments to neurological disease poses challenges, and its application to entirely behaviorally nonresponsive patients has not yet been attempted [59]. Indeed, the development of a QoL instrument in this patient setting faces very serious challenges: patients cannot be interviewed about their values post brain injury; patients cannot complete other validated QoL instruments; and responses to questions are limited to “yes” or “no” [60-62]. Recognizing that these limitations undermine standard methods for QoL instrument development, we will convene an interdisciplinary group of experts (including QoL methodologists, philosophers, neuroscientists, health care workers, and family members of patients who have suffered a serious brain injury) in a two day workshop to address the problem. The purpose of the meeting is (1) to develop an approach to assess overall QoL in behaviorally nonresponsive patients, (2) to develop an approach to assess the health-related QoL in these patients, and (3) to develop strategies to validate these instruments. In developing novel QoL instruments, we hope to gain further insight into the interests and lived experiences of behaviorally nonresponsive patients in order to positively impact their care.

### Project 3: The impact of neuroimaging on families of patients with serious brain injury

Families play an important role for patients diagnosed as vegetative or minimally conscious after serious brain injury. They act as proxy decision makers, assist in providing care, and suffer the emotional strain that accompanies chronic illness and uncertainty [63-67]. Families commonly believe that patients possess conscious awareness despite the lack of any behavioral evidence [68-70]. Neuroimaging may provide insights into residual cognitive function, including covert conscious awareness in some patients, but it is unknown how best to inform families of the risks and benefits of neuroimaging, or to what degree they can comprehend this complex information. The impact of test results on families is also unknown. While positive results may be met with a sense of optimism or even vindication, they also raise the possibility that the patient may be suffering. Negative results may be difficult to understand, as they could indicate that a patient is not aware *or* there has been a failure of the test conditions (e.g., a patient moves too much, has fallen asleep, or cannot concentrate adequately on the experimental tasks). In this case, test results may be a source of confusion or distress for families who believe deeply that the patient is aware.

In this project, we will explore the impact of neuroimaging on the families of behaviorally nonresponsive patients. We seek to gain insight into families’ (1) knowledge of the patient’s medical condition, including their beliefs about the patient’s preserved cognitive function and prognosis, (2) reasons for enrolling the patient in research and their expectations regarding neuroimaging, and (3) experience of research participation and their suggestions as to how study procedures may be improved. We further seek to (4) develop educational materials for families considering neuroimaging for a patient following serious brain injury.

Our first three goals will be addressed in a qualitative interview study using the grounded theory constructivist approach of Charmaz [71]. This approach focuses on understanding participants’ experiences and how they assign meaning to these experiences by gathering rich data through in-depth interviews. English speaking family members who are acting as the surrogate decision maker for patients participating in our neuroimaging research program on serious brain injury at Western University are eligible for this study. Patients must have a diagnosis of vegetative or minimally conscious state and be at least 1 year post-injury. Qualitative sampling is purposive and requires that enough data are generated to sufficiently explore the issues under investigation [72]. The data reaches a point of saturation when no new information or themes are being generated; at this point, interviewing stops. We will interview approximately 30 family members, which should generate sufficient data to reach saturation [72].

Participants will be identified through our program of research on serious brain injury. Typically, family members of patients approach our research program directly via our advertized contact details and first contact is with our CIHR-funded research associate. The research associate will inform potential participants of this qualitative study, obtain verbal consent, and arrange a time for the interview. Written consent will be obtained at the time of the face-to-face interview. Interviews will be conducted by a doctoral student with experience in qualitative interviewing and supervised by a medical sociologist (Webster).

There will be two 90 minute interviews: the first will occur prior to the neuroimaging session, the second will occur after the completion of the neuroimaging study and feedback of study results. The interview guide will map onto the goals of our research while still being flexible enough to allow issues to emerge from the interviews that were not pre-determined by the study team. It will consist of topics beginning with the participant’s relationship with the patient, followed by questions pertaining to his or her beliefs about the patient’s condition and why he or she is participating in neuroimaging studies. All questions are meant to be exploratory and will rely on prompts to allow for differences in perception and experience between participants to emerge during the course of the interview. The interview guide will be pilot tested with at least one participant to ensure that the flow of questions is well ordered and easily understood. Interviews will be digitally recorded and transcribed verbatim and once de-identified will become the text that is analyzed by the team. The transcripts will be imported into a qualitative software program (NVivo 10) that helps organize and retrieve data.

In grounded theory, emergent themes are not just used to explore an issue, but also to construct a cohesive idea or theory about an investigated phenomenon. This analysis emphasizes the connection between theory, concepts, and empirical data through the constant comparative method. Central elements of grounded-theory data analysis include: some form of purposive sampling; simultaneous data collection and analysis that allows for emerging themes to be pursued; identification of social processes within the data; inductive development of abstract theories or categories to explain these processes; comparing, connecting and integrating concepts; and, integration of these concepts or themes into a theoretical framework that describes and explains the phenomenon under study.

The analysis of study data will occur in the following steps: coding, memoing, member checking and theory/model formulation. Codes identify features of the data that are pertinent to the research questions, and organize data into more concise ideas that can be eventually grouped into themes. Open coding is performed to get a more general feel for the content of the data. Axial coding explores relationships between these codes. Selective coding involves integrating these codes or concepts into a core explanatory theory. Four members of the research team will independently read and code the first two interview transcripts. They will then meet to compare their independent analyses and develop a codebook that will be used by the doctoral student in the subsequent analysis. The interviewer’s thoughts and comments throughout data collection will be recorded and analyzed with the goal of focusing thoughts around the emerging concepts. Participants will also be sent a summary of the preliminary analysis. Finally, we will formulate a general theory/model about the nature of participants’ experiences related to serious brain injury and neuroimaging. This will be accomplished through a series of team meetings involving all investigators, in which the relationships between the themes will be summarized. Our analysis will be reflexive by identifying and considering the personal biases of the research team during data analysis. The interview results will be used, in part, to inform and refine the development of a set of educational materials for families considering neuroimaging for a patient following serious brain injury. The interview results will allow us to identify common misconceptions and address them directly in educational materials and to incorporate family member suggestions to improve communication with families.

### Project 4: Ethical issues in the use of neuroimaging in comatose patients with serious brain injury

There are important—and morally relevant—differences between the population of vegetative and minimally conscious patients undergoing neuroimaging and the population of comatose patients now being studied with neuroimaging to map residual cognitive function. The most striking difference is the proximity to injury. In our program of research in serious brain injury at Western University since 2012, the time since injury among vegetative and minimally conscious patients who underwent neuroimaging (n = 24) was a median of 5 years; the median time since injury among comatose patients (n = 12) was 19.5 days. As we have explained, the first days and weeks following a serious brain injury are a time of considerable prognostic uncertainty, and this complicates decisions faced by health care providers and families. The decision whether to continue—or withdraw—LST is prominent in the days and weeks following injury. Serious brain injury is a catastrophic event, and families of recently injured patients are unlikely to have come to terms with the injury or implications for the patient’s functional recovery [69,73]. As a result, families of comatose patients may be particularly vulnerable, fail to understand the patient’s medical condition, and have difficulty comprehending the purpose, harms, and benefits of neuroimaging. Finally, while pilot studies in comatose patients have revealed residual cognitive function, no patient to date has demonstrated covert awareness. As a result, questions related to sentience, communication, and decision making capacity are likely not relevant to these patients—at least not at this moment in their illness trajectory.

Despite the unique challenges posed by conducting neuroimaging in comatose patients following serious brain injury, to the best of our knowledge they remain unaddressed in the ethics literature. In this project, we aim to provide the first sustained ethical analysis of the use of neuroimaging to detect residual cognitive function in comatose patients. Specifically, we will (1) determine whether individual neuroimaging results ought to be shared with the families of comatose patients, and (2) explore potential interactions between neuroimaging results and decisions regarding the use of life-sustaining therapy in comatose patients.

#### ***Subproject 4a: Sharing individual research results with families of comatose patients***

In the first subproject we ask whether and, if so, how individual neuroimaging results ought to be shared with patients’ families. The use of neuroimaging to detect residual cognitive function in comatose patients is experimental. It is conducted with the oversight of a research ethics board and the informed consent of next-of-kin. It is widely accepted that summary research results ought to be shared with participants once the study has been concluded [74,75]. The disclosure of individual research results, however, remains controversial, particularly in this context. In previous work, we argued that individual research results ought to be disclosed if four criteria are fulfilled: (1) disclosure does not seriously undermine the scientific validity of the study; (2) the results are informative and reasonably reliable; (3) the potential benefits of disclosure to the participant outweigh the potential harms; and (4) the participant consents to be informed of the results (Graham et al., submitted). Here we will apply these criteria to the disclosure of neuroimaging results in the intensive care unit context. Key issues will include the informativeness and reliability of neuroimaging results, and the benefits and harms of disclosure. If we conclude that individual results ought to be shared, we will develop a document outlining best practices for disclosure of neuroimaging results in comatose patients.

#### ***Subproject 4b: Neuroimaging and decisions about LST in comatose patients***

In the second subproject we will explore potential interactions between neuroimaging results and decisions to continue or withdraw LST in comatose patients. As we have seen, decisions to withdraw LST may occur within 72 hours of injury, too soon in some cases to have an accurate prognostic picture [26]. A legally authorized proxy decision maker may refuse or withdraw any medical care—be it mechanical ventilation or artificial nutrition and hydration—inconsistent with the patient’s prior expressed wishes or values [76]. Despite this, proxy decision makers may be very reluctant to withdraw artificial nutrition or hydration [27]. As a result, proxy decision makers seeking to prevent an undesired neurological outcome may feel compelled to act in the face of prognostic uncertainty due to a perceived “window of opportunity” to withdraw treatment while the patient remains dependent on a ventilator. We will argue that ethical decisions regarding LSTs should both respect the prior expressed wishes and values of the patient and be based on reasonably obtainable and reliable prognostic information. In some cases, a grave prognosis may be evident soon after injury based on clinical examination and structural neuroimaging. In other cases, neuroimaging to map residual cognitive function may provide important clues to a comatose patient’s prognosis [23,77]. If the patient’s prior expressed wishes involve not wanting to “end up vegetative” or otherwise profoundly neurologically disabled, we will argue that a valid proxy decision must take account of available prognostic information. When the prognosis is unclear and functional neuroimaging is available, there is a prima facie obligation to build a clearer prognostic picture before a decision regarding LST is undertaken.

### Ethical considerations

Subproject 1b (Demonstrating the feasibility of decision making capacity assessment using neuroimaging) and project 3 (The impact of neuroimaging on families of patients with serious brain injury) involve research on human participants. Both studies have been reviewed and approved by the University of Western Ontario Research Ethics Board for Health Sciences Research Involving Human Subjects (#100070 and #104684). Informed consent will be obtained from all study participants.

## Discussion

Serious brain injuries place an enormous burden on patients, families, and the healthcare system. Currently, 1.4 million Canadians are living with the effects of brain injury, with 50,000 new cases occurring each year. Patient outcome after serious brain injury is highly variable. Following a period of coma lasting days or weeks, some patients make a good recovery, while others progress into a vegetative or minimally conscious state. As it is difficult to predict who will make a good recovery after serious brain injury, families and physicians are forced to make treatment decisions in the face of uncertainty. Further, the diagnosis of vegetative and minimally conscious states is itself difficult, with error rates as high as 43%. Recent advances in neuroimaging allow for the detection of intact brain function that cannot be found by routine bedside examination. Neuroimaging offers the prospect of improved prediction of outcome and increased diagnostic accuracy. But neuroimaging after serious brain injury poses profound ethical questions that must be answered before it can be applied widely.

Each of our four projects addresses an important ethical issue, and is designed to both add to our knowledge of the ethics of neuroimaging and contribute to ethical practice. Project 1 seeks to develop an approach to the assessment of decision making capacity in behaviorally nonresponsive patients using neuroimaging. This work will contribute to the broader literature on capacity assessment in patients with neurological impairments. If it is applied to the patient setting successfully, it will allow patients with preserved capacity the opportunity to participate in their own care decisions. Project 2 will argue that behaviorally nonresponsive patients with covert awareness are very likely sentient and will explore the application of an ethics of welfare to these patients. This work will contribute to the literature on the ethics of welfare in bioethics. Our efforts to develop quality of life instruments for behaviorally nonresponsive patients represent the first instance of systematically exploring the subjective experiences of these patients, and offer the prospect of improving patient quality of life. Project 3 explores the impact of neuroimaging on the families of patients after serious brain injury. Little is currently known, and the interviews will further our understanding of family knowledge, family expectations, and the impact of neuroimaging on these views. The educational materials developed will ensure that families considering neuroimaging are better informed. Project 4 explores the use of neuroimaging in a new setting, namely comatose patients within days of serious brain injury. To our knowledge, this will be the first sustained ethical analysis of these issues. Our work will have an impact on how families are informed about neuroimaging results and seeks ultimately to improve decisions regarding life sustaining therapy.

## Abbreviations

CIHR: Canadian Institutes of Health Research; EEG: Electroencephalography; fMRI: Functional magnetic resonance imaging; LST: Life sustaining therapy; QoL: Quality of life.

## Competing interests

The authors declare that they have no competing interests.

## Authors’ contributions

AMO and CW contributed to the conception and design of the overall protocol. AP and LN contributed to the conception and design of project 1b, KS contributed to the conception and design of project 2c, and LGL and FW contributed to the conception and design of project 3. AP wrote the initial draft of the background and project 1, MG wrote the initial draft of project 2, FW wrote the initial draft of project 3, and CW wrote the initial draft of project 4. CW led the writing of subsequent versions. All authors commented on sequential drafts and approved the final version.

## Pre-publication history

The pre-publication history for this paper can be accessed here:

http://www.biomedcentral.com/1472-6939/15/41/prepub
